# Anatomical Specializations for Nocturnality in a Critically Endangered Parrot, the Kakapo (*Strigops habroptilus*)

**DOI:** 10.1371/journal.pone.0022945

**Published:** 2011-08-10

**Authors:** Jeremy R. Corfield, Anna C. Gsell, Dianne Brunton, Christopher P. Heesy, Margaret I. Hall, Monica L. Acosta, Andrew N. Iwaniuk

**Affiliations:** 1 Department of Neuroscience, University of Lethbridge, Lethbridge, Alberta, Canada; 2 Institute for Natural Sciences, Massey University, Auckland, New Zealand; 3 Department of Anatomy, Midwestern University, Glendale, Arizona, United States of America; 4 Department of Physiology, Midwestern University, Glendale, Arizona, United States of America; 5 Department of Optometry and Vision Science, University of Auckland, Auckland, New Zealand; Lund University, Sweden

## Abstract

The shift from a diurnal to nocturnal lifestyle in vertebrates is generally associated with either enhanced visual sensitivity or a decreased reliance on vision. Within birds, most studies have focused on differences in the visual system across all birds with respect to nocturnality-diurnality. The critically endangered Kakapo (*Strigops habroptilus*), a parrot endemic to New Zealand, is an example of a species that has evolved a nocturnal lifestyle in an otherwise diurnal lineage, but nothing is known about its' visual system. Here, we provide a detailed morphological analysis of the orbits, brain, eye, and retina of the Kakapo and comparisons with other birds. Morphometric analyses revealed that the Kakapo's orbits are significantly more convergent than other parrots, suggesting an increased binocular overlap in the visual field. The Kakapo exhibits an eye shape that is consistent with other nocturnal birds, including owls and nightjars, but is also within the range of the diurnal parrots. With respect to the brain, the Kakapo has a significantly smaller optic nerve and tectofugal visual pathway. Specifically, the optic tectum, nucleus rotundus and entopallium were significantly reduced in relative size compared to other parrots. There was no apparent reduction to the thalamofugal visual pathway. Finally, the retinal morphology of the Kakapo is similar to that of both diurnal and nocturnal birds, suggesting a retina that is specialised for a crepuscular niche. Overall, this suggests that the Kakapo has enhanced light sensitivity, poor visual acuity and a larger binocular field than other parrots. We conclude that the Kakapo possesses a visual system unlike that of either strictly nocturnal or diurnal birds and therefore does not adhere to the traditional view of the evolution of nocturnality in birds.

## Introduction

Living in a scotopic, or low light, environment poses significant challenges for the visual system. In contrast to photopic, or well-illuminated, environments where the chances of the retina capturing a photon are extremely high, in scotopic environments, light levels are typically about a million times lower [1] and the visual system relies on various specializations. As a result, the visual systems of animals that live in scotopic environments have evolved in one of two ways. Firstly, they can evolve mechanisms to increase the sensitivity of the eye to light. Examples of this include increasing the size of the eye, the size of the cornea relative to the axial length of the eye, and/or density and type of photoreceptors in the retina [2]–[5]. In addition, increasing orbit convergence and binocular visual field overlap can increase light capture by increasing the probability of capturing a quantum of light within the region of overlap [6]–[9]. Alternatively, animals can decrease their emphasis on the visual system and enhance the sensitivity of other sensory systems to provide equivalent information about their environment. Kiwi (*Apteryx* spp.), moles and mole-rats are all prime examples of this second strategy. These species have relatively small eyes and visual brain regions, but greatly enlarged somatosensory systems and tactile specialisations in their extremities [4], [10]–[14]. Thus, shifting from a diurnal to a nocturnal lifestyle can either be associated with the enlargement of the visual system to enhance light sensitivity or the reduction of the visual system combined with the enlargement of other sensory systems.

Although in fishes and mammals there are numerous examples of both strategies, the extent to which individual species evolve one strategy or the other in birds is not well understood. Nocturnality has evolved multiple times in otherwise diurnal avian lineages [15], [16] and, in the case of owls diurnality has evolved several times within an otherwise nocturnal lineage [17]. One of the most profound shifts in activity pattern from diurnality to nocturnality has occurred in the critically endangered New Zealand parrot, the Kakapo (*Strigops habroptilus*), a parrot unlike any other in many aspects [18]. It is the largest parrot worldwide; it is nocturnal, flightless and an obligate herbivore with a strong body-odour [19]–[21]. Its nocturnal lifestyle, combined with its owl-like facial ruff, earned this species the moniker ‘owl parrot’ [22], [23]. To successfully conserve this enigmatic species, there is a strong need to understand its sensory abilities and unique nocturnal lifestyle.

Pettigrew (1978) suggested that the Kakapo could have visual specializations similar to that of owls based on its nocturnal activity pattern and the presence of a facial ruff. Hall et al. (2009) further suggested that the optic foramen size fell well within the range of nocturnal birds, although analyses of the eyes or brain of the Kakapo have not been carried out. In this study, we provide the first detailed comparative examination of the size and shape of the brain as well as the eyes and orbits of this enigmatic species. We compare our data with closely related parrots such as the Kea (*Nestor notabilis*), a sister taxon to the Kakapo [24], as well as more distantly related parrots.

Because very little is known about retinal morphology in parrots, the retinal morphology of the Kakapo is compared with that of two diurnal parrot species as well as the nocturnal Barn Owl (*Tyto alba*) and the diurnal chicken (*Gallus domesticus*). Much is known about the visual systems of both Barn Owls and chickens [25]–[28] and they exhibit a retinal morphology that is typical of nocturnal and diurnal birds, respectively. By comparing retinal morphology across these species, we will be able to determine whether the Kakapo has a retina typical of parrots or one more similar to that of nocturnal birds, like the Barn Owl.

In principle, there are two expected outcomes. If the Kakapo has enhanced light sensitivity in a comparable fashion to other nocturnal birds, then from an anatomical perspective it should have relatively large eyes, an eye shape with a larger cornea relative to the axial length of the eye, more rods in the retina and more convergent (i.e., similar facing) orbits [2], [29]–[38], all of which function to increase light gathering in dim environments. Similarly, if the Kakapo has stereoscopic abilities comparable to that of nocturnal owls, the brain of the Kakapo should have: 1) an enlarged Wulst [39]; 2) a markedly reduced optic tectum [35], [40], [41]; and 3) correspondingly smaller forebrain targets of the optic tectum [40]. Alternatively, if the Kakapo has diminished its reliance on vision, it should have relatively small eyes, few rods in its retina, little change in corneal diameter and a relatively small optic tectum as well as its corresponding forebrain targets. Our data suggests that the Kakapo has undergone profound changes to the morphology of its visual system. Some features of the Kakapo visual system are typical of nocturnal birds, the brain morphology is vastly different from that of other parrots and other aspects of the visual system are intermediate with respect to diurnal and nocturnal birds.

## Results

### Brain Morphology

**Figure 1:**
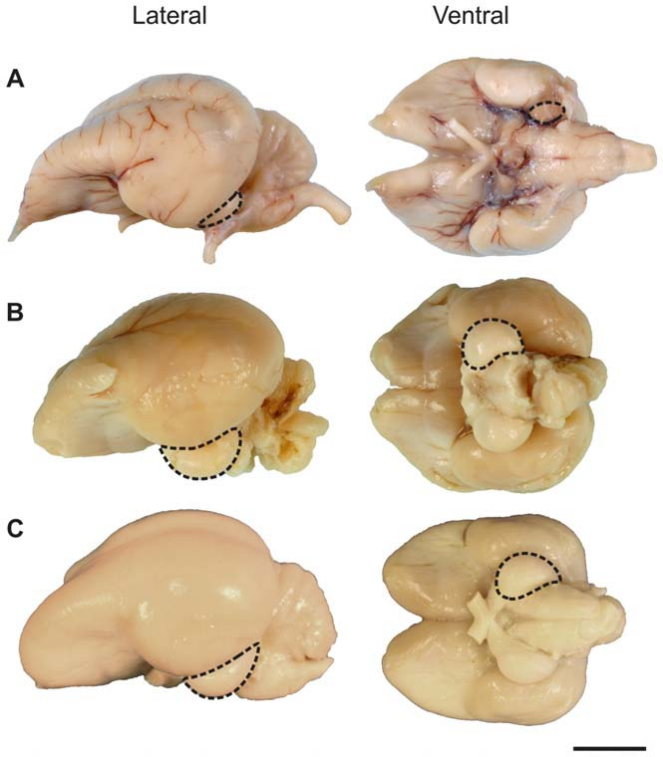
Photos of the brains of three species of parrots. A, the Kakapo (*Strigops habroptilus*); B, Kea (*Nestor notabilis*); and C, Sulphur-crested Cockatoo (*Cacatua galerita*). The dotted lines outline the left optic lobe of all three species. Abbreviations refer to the following: OB, olfactory bulbs; W, Wulst; Cb, cerebellum; and TeO, optic lobe. Scale bar = 10 mm.

The adult Kakapo brain has a length of 5.2 cm and a width of 3.5 cm. While both the olfactory bulbs and Wulst are prominent, the optic lobes are extremely small and partially obscured by the lateral aspect of the cerebral hemispheres (Fig. 1A). In contrast, the Kea brain is relatively wider than the Kakapo (Fig. 1B) with a length of 4.2 cm and a width of 3.8 cm. The olfactory bulbs were damaged and, as a result, missing in the extracted Kea brain and the brainstem slightly damaged; however, the optic lobes were prominent, as they are in other parrots (Fig. 1C).

### Brain Volumetrics

**Figure 2:**
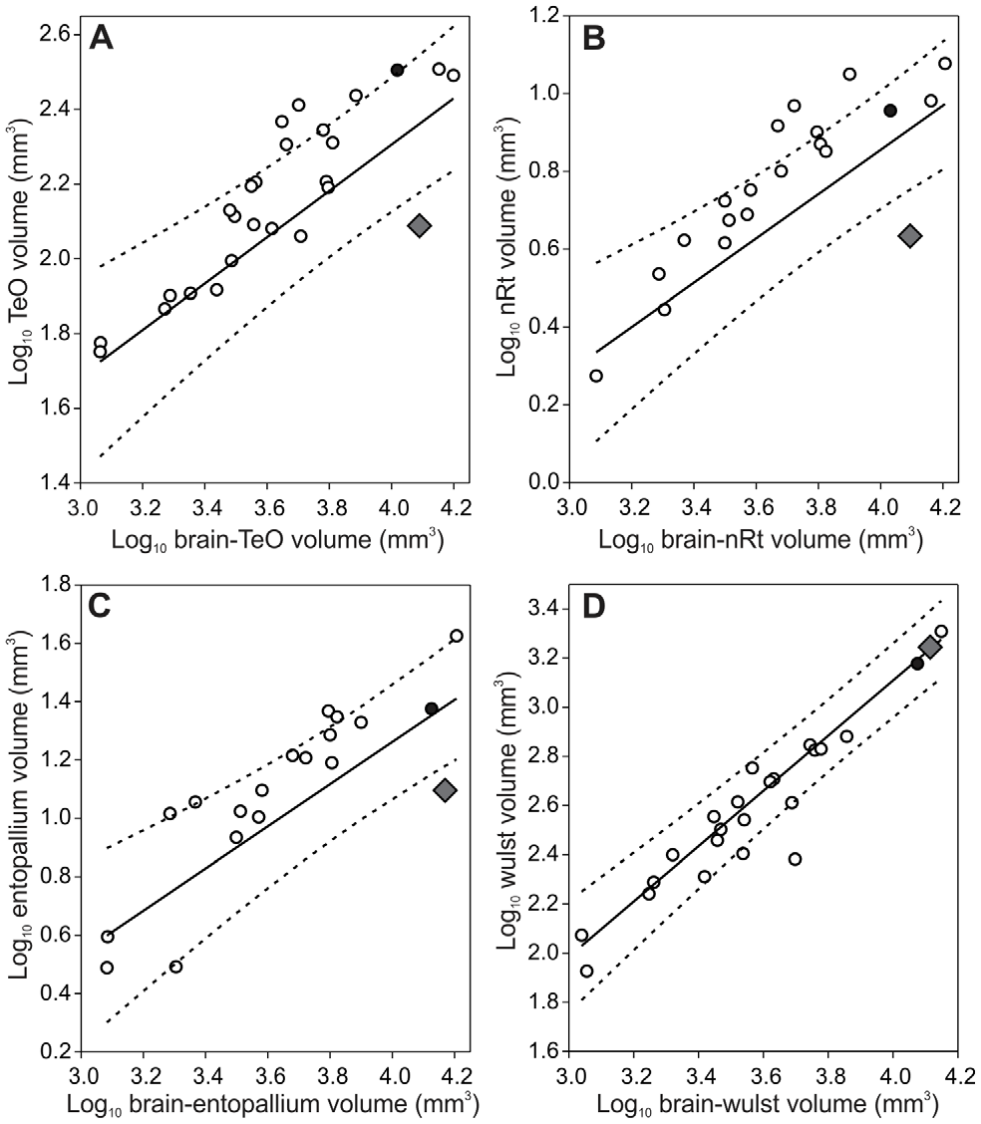
Scatterplots of each of the four visual brain regions measured against total brain volume. A, optic tectum (TeO); B, nucleus rotundus (nRt); C, entopallium; and D, Wulst. All measurements in mm^3^. The solid lines indicate the least-squares linear regression lines and dotted lines indicate the phylogeny-corrected 95% confidence interval. The diamond represents the Kakapo (*Strigops habroptilus*), the filled circle represents the Kea (*Nestor notabilis*) and open circles represent other parrot species included in the analyses.

As suggested by the external appearance of the Kakapo brain, the optic tectum (TeO) is significantly reduced in size relative to the total size of the brain (Fig. 2A). The same is also true of the other two tectofugal regions, nucleus rotundus (nRt) and entopallium; both of them are significantly smaller in the Kakapo compared to other parrots, including the Kea (Fig. 2B,C). In contrast, the Wulst, the one region of the thalamofugal visual pathway that we could measure, did not show a reduction in size (Fig. 2D), and is similar in relative size across most parrots examined.

### Optic foramen

**Figure 3:**
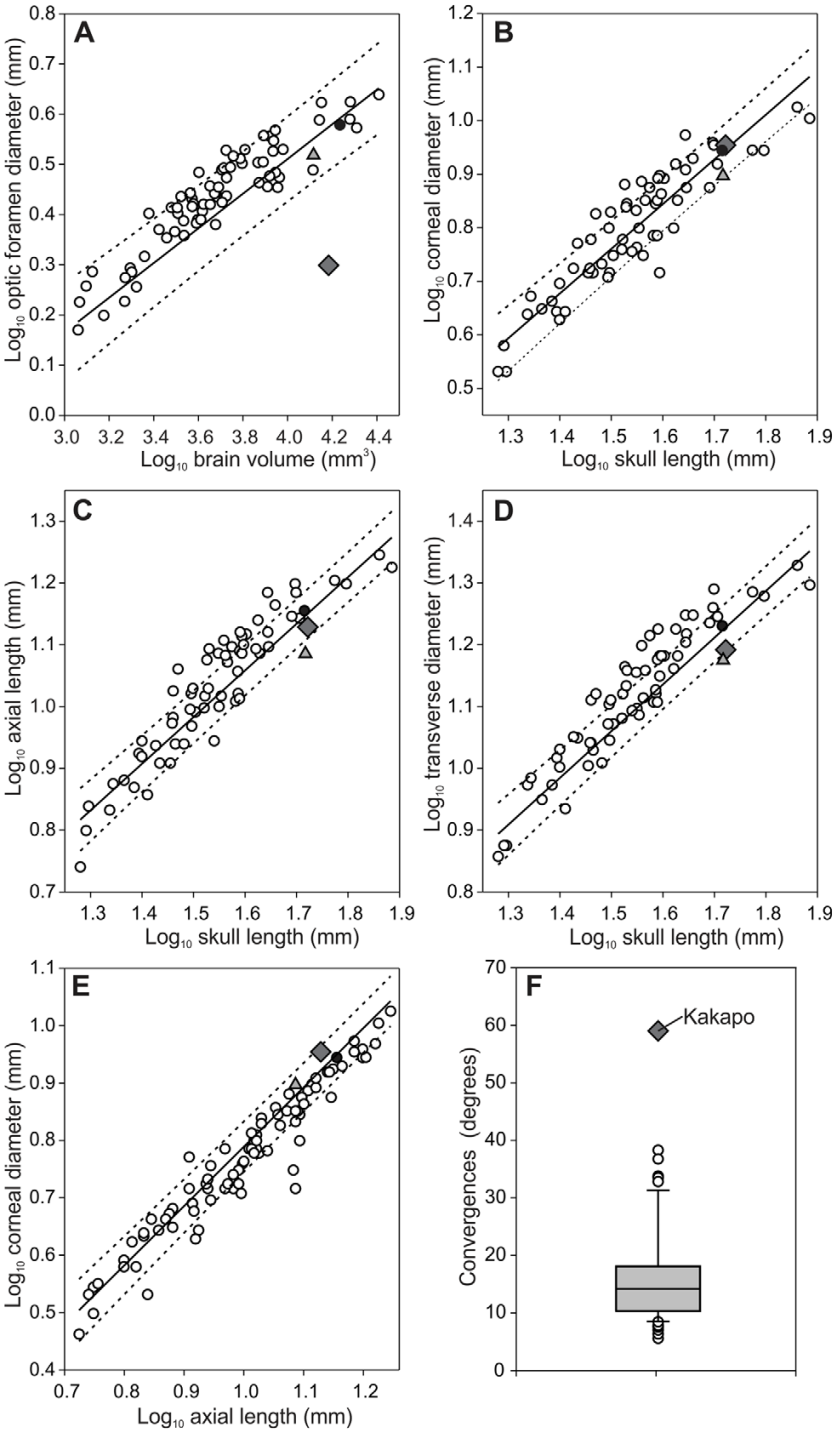
Scatterplots for each of the parameters measured from the eye. A, optic foramen diameter, B,C,D, eye size, and E, eye shape. Kakapo (diamond), Kea (filled circle), Kaka (triangle) and all other parrots (open circles). The solid lines indicate the least-squares linear regression lines and dotted lines indicate the phylogeny-corrected 95% confidence interval. F, is a boxplot of orbit orientation (in degrees) measured in the Kakapo and 64 other parrot species. The box plot shows the smallest observation (sample minimum), lower quartile (Q1), median (Q2), upper quartile (Q3), and largest observation (sample maximum). Outliers are shown as open circles and also a diamond for the kakapo.

The optic foramen of the Kakapo is significantly smaller than that of other parrots, regardless of what scaling measure is examined and whether or not phylogeny is taken into account (Fig. 3A) and is within the range of other nocturnal birds [42]. In fact, the diameter of the Kakapo's optic foramen is similar to that of the Red-rumped Parrot (*Psephotus haematonotus*), a species with a body mass 1/25^th^ and a brain volume 1/8^th^ that of the Kakapo [42]. The small size of the Kakapo's optic foramen is also apparent when it is contrasted with both the Kea and the Kaka (*Nestor meridionalis*; Fig. 3A). Both the Kea and Kakapo share a similar skull length and brain volume, however, the Kea's optic foramen is two times larger than that of the Kakapo.

### Eye size and shape and orbit orientation

Relative to head length and brain volume, corneal diameter, axis length and transverse diameter of the Kakapo eye were not different from that of other parrots (Fig. 3B–D). The shape of the Kakapo eye, as described by a plot of corneal diameter against axial length, is also within the range of other parrots (Fig. 3E). The Kakapo also has significantly more convergent orbits than other parrots (Fig. 3F). Whereas most parrots have orbital convergence values of 5–25°, the Kakapo's orbital convergence is more than double (59°) the average (16.3°±9.2°). Thus, the orbits of the Kakapo are more convergent (i.e., front facing) than any other parrot.

### Retinal anatomy

**Figure 4:**
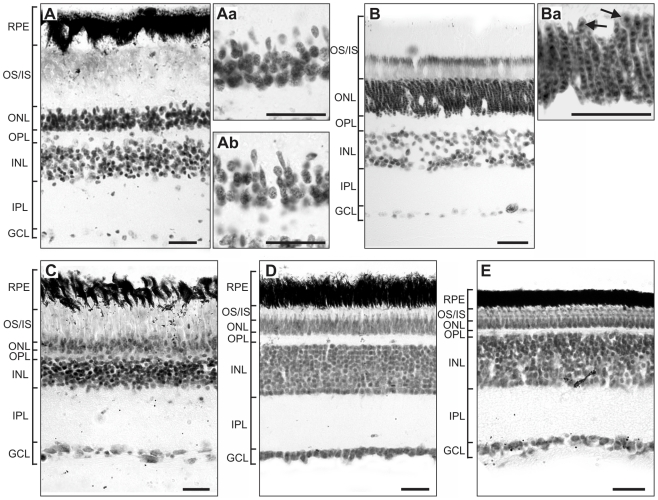
Photomicrographs of transverse sections through the retina of five species of birds. A, Kakapo (*Strigops habroptilus*), B, Barn Owl (*Tyto alba*), C, Sulphur-crested Cockatoo (*Cacatua galerita*), D, Eastern Rosella (*Platycercus eximius*), and E, Chicken (*Gallus gallus domesticus*). Aa and Ab are photomicrographs of peripheral and central photoreceptors respectively in the kakapo retina at 100 times magnification. Ba is a higher magnification image of the photoreceptors in the Barn Owl. The arrows indicate cone photoreceptor cells. Retinal tissue was stained with cresyl violet. Abbreviations are as follows: RPE, retinal pigmented epithelium; OS/IS, outer segment/inner segment; ONL, outer nuclear layer; OPL, outer plexiform layer; INL, inner nuclear layer; IPL, inner plexiform layer; GCL, ganglion cell layer. Scale bar = 25 µm.

In the Kakapo, the overall length of the photoreceptors is larger than the other species examined in this study. This is because photoreceptor's outer and inner segments are longer than in diurnal species studied here (Fig. 4, Table 1). The Barn Owl has an extreme specialisation for night vision; they have elongated rods and possibly double cone photoreceptor cells ([43], Fig. 4Ba). Neither elongated rods nor putative double cones were observed in the Kakapo photoreceptors either in central or peripheral retina (Fig. 4Aa, Ab). The diurnal species had a relatively thin photoreceptor layer, however, in the Cockatoo (*Cacatua galerita*) the outer/inner segment length is comparable to nocturnal species. The outer nuclear layer (ONL), formed by the rod and cone photoreceptor nuclei, has the highest relative thickness in the Barn Owl and is relatively thin in the Chicken and Eastern Rosella (*Platycercus eximius*) (Fig. 4B, D, E, Table 1). The relative thickness of the ONL in the Kakapo and Cockatoo are similar to one another and have an intermediate thickness (Fig. 4A, C, Table 1). Also, the relative thickness of the inner nuclear layer (INL), which contains the cell bodies of the amacrine and bipolar cells, is much thinner in the Kakapo compared to the Chicken and Eastern Rosella and similar to that of the nocturnal species, the Barn Owl. Finally, although the condition of the eyes from the Kakapo specimen were not properly fixed for analyses of retinal ganglion cell density or detailed structural analyses of the retinal layers and no distinction between ganglion cells and displaced amacrine cells was made, we were able to estimate ganglion cell layer (GCL) density, as shown in Figure 4. The staining of the GCL of the Kakapo revealed relative few cells compared to the other species examined (Fig. 4, Table 1). The cell density in the GCL was similar to that of the Barn Owl.

**Table 1:** Shows average thickness for central and peripheral retina and retinal layers in diurnal and nocturnal species.

	total	OS IS	ONL	INL	GCL	OS-IS/total	ONL/total	INL/total	GCL/total	GCL cells/mm
Kakapo central	214.34	99.59	35.01	34.41	10.03	0.46	0.10	0.16	0.05	89
Kakapo periphery	211.215	101.25.70	22.14	34.1	11.86	0.48	0.10	0.16	0.06	87
Barn owl central	178.42	**73.97**	**28.81**	29.97	6.89	**0.41**	0.16	**0.17**	0.04	**82**
Barn owl peripheral	170.71	70.12	29.15	31.81	6.03	0.41	0.17	0.19	0.04	96
Chick central	235.60	25.52	13.04	101.19	19.59	0.11	0.06	0.43	0.08	354
Chick peripheral	234.53	27.97	12.15	107.39	15.39	0.12	0.05	0.46	0.07	348
Cockatoo central	158.43	54.80	19.55	**36.84**	14.59	0.35	**0.12**	0.23	0.09	124
Cockatoo peripheral	137.03	46.40	16.41	31.50	13.46	0.34	0.12	0.23	0.10	98
Rosella central	**232.89**	28.88	17.30	55.94	15.31	0.12	0.07	0.24	0.07	285
Rosella peripheral	225.21	42.69	21.30	62.72	15.11	0.19	0.09	0.28	0.07	333

Values are expressed in micrometers. Highlighted values indicate best similarities with the kakapo retina. The Barn Owl is representative of a nocturnal species and the Chicken, Cockatoo and Eastern Rosella diurnal species [17], [22]. Abbreviations: total: total thickness of the retina (microns), OS IS: outer segment/inner segment, ONL: outer nuclear layer, INL: Inner nuclear layer, GCL: Ganglion cell layer. GCL cells/mm indicates the number of cells per mm in the GCL.

## Discussion

In general, a shift from a diurnal to nocturnal lifestyle should entail changes to the visual system that either enhance light sensitivity or decrease the relative importance of vision [5]. Instead of either of these extremes, the Kakapo has a unique combination of traits including a reduction in the relative size of the tectofugal pathway and optic nerve in conjunction with a morphological appearance of the retina that shows features of both nocturnal and diurnal birds. This interesting combination of traits speaks to the Kakapo's unusual phylogenetic position as one of only two species to evolve nocturnality in an otherwise entirely diurnal group, necessitating comparisons both within parrots and with unrelated nocturnal birds. The Kakapo has convergently oriented orbits common for nocturnal vertebrates, and an eye size and shape that is within the range of the diurnal parrots but is also within the range of other nocturnal birds, including the nightjars, nighthawks, and owls [2]. These traits indicate that the Kakapo likely has a larger binocular visual field, which could confer enhanced light capture by increasing the quantum catch probability within the expanded region of overlap (e.g. [7], [44]). In addition, the paucity of retinal ganglion cells compared to other parrots could be indicative of relatively poor visual acuity [33], [42], [45], [46].

Orbit orientation is correlated with the amount of binocular overlap in the visual field of both birds and mammals [33], [45], [46]. The significantly greater amount of orbital convergence in the Kakapo could therefore be taken as an indication of a wider binocular visual field, as predicted by Pettigrew (1978). The orientation of the orbits is not, however, solely responsible for the width of the binocular field. Indeed, eye movements make a significant contribution to the shape of the visual fields of many birds [35]. Thus, the extent to which we can infer the degree of binocularity during various activities in the Kakapo is limited. Similarly, it is difficult to comment on the suggestion [47], [48] that the Kakapo has stereoscopic abilities (i.e., depth perception) similar to that of owls. Unlike owls, the Kakapo does not have an enlarged Wulst, but Wulst hypertrophy is not necessarily a robust predictor of either the binocular visual field or stereopsis [33], [49]. Determining these features of the Kakapo visual system will depend on behavioral testing because neurophysiological studies are unlikely to be feasible in such a highly endangered species.

The sensitivity and acuity of the Kakapo's visual system can be inferred from our data. In most tetrapods, eye size and shape varies according to activity pattern. In general, nocturnal species tend to have broader corneas, relative to the axial length of the eye, than either crepuscular or diurnal species [2], [50]. The purpose of these changes in eye size and shape is to increase the sensitivity of the eye. Corneal diameter is associated with the light gathering ability of the eye. The axial length of the eye is associated with visual acuity; the longer the axial length, the larger the projected image on the retina becomes [5], [51], [52]. An eye shape with a large corneal diameter relative to the axial length of the eye is typical of nocturnal birds, including owls, nightjars and nighthawks, and the oilbird [2]. Although the size and shape of the Kakapo eye is within the range of other parrots, the Kakapo is also within an area of overlap with many nocturnal birds [2]. Therefore, we suggest that the eye shape of the Kakapo is consistent with the typical nocturnal eye shape of birds. Based on eye morphology alone, we would therefore predict that the Kakapo has enhanced visual sensitivity, with concomitantly poor visual acuity.

Both the enhanced sensitivity and poor acuity of the Kakapo, relative to other parrots, are reinforced by the structure of the retina. The Kakapo retina is characterized by a broader photoreceptor layer and an increased length of the outer and inner segment (Table 1). The outer segment of photoreceptors is the area where the photopigment is located and in the inner segment the metabolic and biosynthesis of molecules for the outer segment occur [53]. Thus, increased outer and inner segment length may suggest increased retinal sensitivity. The histological analysis does not reveal specialised photoreceptors cells, however, the moderately thick outer nuclear layer in the Kakapo and the presence of round nucleus located in the most outer part of the ONL, suggestive of cone, indicates that rods and cones are well represented in the retina as in other nocturnal birds [54]. A narrower inner nuclear layer and fewer ganglion cells likely reflect a strategy for increasing retinal sensitivity, although with very poor resolving power [55]. A relatively small number of retinal ganglion cells is also supported by the small size of the Kakapo's optic foramen. The optic nerve, which passes through the optic foramen, is largely comprised of retinal ganglion cell axons. A smaller optic foramen therefore reflects few retinal ganglion cells and is typical of nocturnal species [42]. More photoreceptors per retinal ganglion cell, referred to as increased retinal summation, would then provide enhanced light sensitivity, but poor visual acuity, similar to other nocturnal birds [11], [54]. Based on our measurements of the optic nerve and examination of retinal sections, it would appear that the Kakapo has the requisite morphology of a bird with enhanced low light (mesopic) vision.

The morphology of the Kakapo's brain also yields insight into its visual abilities. Parrots possess relatively small visual regions [40], [41], although the Kakapo has taken this reduction in the tectofugal pathway to an extreme. In fact, apart from kiwi [11], [35], the Kakapo appears to have the smallest tectofugal brain regions of any bird examined to date. This reduction in the visual system in the Kakapo is not, however, universal. The Wulst, the telencephalic target of the thalamofugal pathway, is similar in size to that of other parrots and not enlarged as it is in owls or some caprimulgiforms [39], [41]. The huge reduction in size of the tectofugal pathway combined with no change in Wulst volume strongly suggests a decreased reliance on vision in the Kakapo in a similar fashion to what has occurred in Kiwi [11], [13]. The evolution of flightlessness, folivory and nocturnality on a largely predator-free island may have reduced the Kakapo's reliance on vision in favor of enhancing other sensory modalities [21].

Overall, we conclude that the Kakapo has a unique visual system unlike that of other parrots or any other bird examined to date. The Kakapo is a highly unusual animal that evolved nocturnality in the context of its phylogenetic background as a parrot, and as such it almost certainly had a diurnal ancestor. Therefore, in order to interpret the suite of nocturnal characteristics exhibited by the Kakapo, we must compare it to both parrots and nocturnal birds. Indeed, we can see that the Kakapo possesses traits consistent with nocturnal birds, including owls (retina, eye size and shape and orbit orientation), caprimulgiforms (eye size and shape), and kiwi (brain morphology), and also diurnal birds (eye size and shape). Based on this suite of traits, the Kakapo likely has somewhat reduced its overall reliance on vision. However, its visual abilities are characterized by the larger binocular visual field, enhanced low light sensitivity and poor visual acuity usual for nocturnal birds. In doing so, the Kakapo breaks the dichotomy typical of the evolution of nocturnality in birds and mammals and illustrates that the visual system can evolve in a mosaic rather than a strictly concerted fashion by exhibiting individual nocturnal traits found in a variety of unrelated nocturnal birds.

## Materials and Methods

### Ethics Statement

All specimens were provided to us dead by conservation authorities, wildlife veterinarians and museum staff and thus approval was not required by an institutional ethics committee to undertake this research. Specimens were obtained from the New Zealand Department of Conservation, the Kakapo Recovery Group, Massey University, and the National Museum of Natural History (Washington, DC).

### Specimens

A Kakapo, from the former Fjordland population, was obtained post-mortem from the Auckland Zoo with the permission of the New Zealand's Department of Conservation and the Kakapo Recovery Group. The specimen was processed eight hours post-mortem and the brain and eye were immersion-fixed and stored in 4% paraformaldehyde (PFA) in phosphate buffered saline (PBS). An adult male Kea specimen was received by Massey University's Institute of Veterinary, Animal and Biomedical Sciences in February 2009 and immersion-fixed in 4% PFA in PBS by a Massey University veterinarian. Unfortunately, the Kea brain had a badly damaged hindbrain and cerebellum, so the description of the Kea brain and its volumetrics are limited to the optic lobe and telencephalon. The eyes of an Eastern Rosella (*Platycercus eximius*) and a Sulphur-crested Cockatoo (*Cacatua galerita*) were obtained from birds culled in a regional pest management programme in Auckland, New Zealand. Tissues were immersion-fixed in 4% paraformaldehyde (PFA) in phosphate buffered saline (PBS) for 30 minutes, washed several times in PBS and transferred into 30% sucrose until sectioned.

Before sectioning, the brains were photographed using a Nikon D2Xs digital camera with a 105 mm f/2.8D AF Micro-Nikkor lens. To compare brain anatomy, all specimens were processed in a similar way: both brains were cut sagittally with a razor blade and each half was cryoprotected in 30% sucrose in 0.01 M PBS (about 10 days). The brains were embedded in gelatin and sectioned in the sagittal plane on a sliding freezing stage microtome at a thickness of 45 µm. The sections were collected in PBS and subsequently mounted onto subbed slides, stained with cresyl violet, dehydrated and coverslipped with DePeX (SERVA GmbH).

### Volumetric Measurements

We measured four regions, all of which are involved in visual processing: optic tectum (TeO), nucleus rotundus (nRt), entopallium and the Wulst. The optic tectum is the primary target of retinal ganglion cells in the avian brain [56], largely projecting to the thalamic target nRt. nRt, in turn, projects to the entopallium of the telencephalon and together these three brain regions comprise the tectofugal pathway [57]. The Wulst is the telencephalic target of a separate visual pathway: the thalamofugal [57]. The Wulst is greatly enlarged in owls and some other families [33], [39], [41] and plays a key role in stereoscopic vision in these taxa [47], [48], [58], [59], although its role in modulating stereopsis in other taxa has been debated [33], [49].

In terms of delineating these four regions, we adhered to descriptions in the literature as well as several stereotaxic atlases [60]–[65]. As with previous studies, we defined the optic tectum as all laminated layers of the tectum, excluding the optic tract [33], [39], [66], [67]. The nRt is readily defined by the presence of large, intensely Nissl stained cells of low density relative to adjacent structures and the borders of entopallium were defined by the description of Nissl stained tissue outlined in [68], [69]. Volumetrics of other parrot species were obtained from previously examined tissue [40], [66] and the literature [70], [71]. Details of the brain region volumes and sample sizes are provided in Table S1.

Brain sections were imaged using a Leica stereomicroscope, and the images subsequently loaded into Amira (v 5.2, Mercury Computer Systems, San Diego, CA, US) for alignment and modelling. Photos were taken of every second brain section in the Kakapo and Kea. Total brain volume and volume of the individual brain regions were calculated while labelling each section in Amira individually and according to the locations of the different brain areas. The outlines of each brain region obtained in Amira were exported as a series of TIFF files. In these, a given region is filled in black against a white background. These TIFF stacks were then used for volumetric estimates of each region using ImageJ (National Institutes of Health, USA, http://rsb.info.nih.gov/ij/). Each image was then analyzed to obtain the cross-sectional area of the brain region. To calculate the total volume of the region the cross-sectional areas were added for each brain region and then multiplied by the slice thickness and the number of sections between stack slices.

Shrinkage factors were calculated by comparing brain volumes prior to processing with brain volumes calculated by measuring serial sections on the slides. The areas of entire coronal sections were measured throughout the brain and multiplied by section thickness (45 µm) and the sampling interval. The difference between this measurement and the original brain volume yielded a shrinkage factor (Kakapo = 1.23; Kea = 1.25), which was subsequently applied to all of our measurements as in [66], [67], [70], [72]–[74].

### Morphometrics

Brain volume, skull length, orbit dimensions and cross-sectional areas of the optic foramen and foramen magnum were made from specimens at the National Museum of Natural History (Washington, DC) and were measured in 199 specimens representing 83 parrot species (Table S1). Brain volumes were measured from the skull by filling the endocranial cavity via the foramen magnum with a 50∶50 mixture of sizes 8 and 9 lead shot. This procedure provides an unbiased estimate of brain volume [75], and was used as an independent variable in examining relative optic foramen and eye size. All linear measurements were made with dial calipers to the nearest 0.1 mm. Skull length was measured from the midpoint of the nasofrontal hinge to the caudal-most point of the braincase. Optic foramen diameter was measured as in Hall et al. (2009). Finally, we measured the minimum and maximum diameters of the foramen magnum and then estimated the cross-sectional area using the formula for an ellipse.

### Orbit Orientation

Morphometric data on orbit orientation were collected from 138 specimens at the National Museum of Natural History (Washington, DC), representing 65 species, including two Kakapo skulls (Table S1), following the same protocol outlined in Iwaniuk et al. (2008). Briefly, three-dimensional coordinate data were collected for the six landmark points on the skull with a MicroScribe-3DX coordinate data stylus (Immersion Corp., San Jose, CA). Each specimen was mounted on an elevated clay base so that all coordinate data could be collected in a single series [76]. The six landmark points are as follows: 1) the anterior-most point of the beak; 2) that point where the internasal suture meets the inter-premaxillary suture; 3) the posterior-most projection on the skull, at the superior-most portion of the occipital complex; 4) the mid-point on the quadratojugal bar; 5) the point on the orbital margin that is directly opposite and furthest from the midpoint on the quadratojugal bar; and 6) the central point of the lacrimal bone. Orbit convergence was calculated from these coordinate data following a standard trigonometric function for dihedral angle computation [77]. A macro for this calculation is available in Heesy (2003) and further details are provided in Iwaniuk et al. (2008). The macro calculates the angle of convergence for a single orbit. For consistency we have used convergence as an alternative to calculating inverse of the angle of divergence [33], [45], [78]. Multiplying the angle by two yielded the total (or bilateral) convergence of both orbits.

### Eye and Retina

Data on eye size of the Kakapo and data from 117 other parrots species are from Ritland (1982) and Hall and Ross (2007). Three parameters were examined: corneal diameter, eye axial length and transverse diameter. Axial length of the eye refers to the medio-lateral distance from the centre of the cornea to the medial-most portion of the eyeball, just anterior to the exit of the optic nerve, whereas transverse diameter is the width across the eye at the maximum protrusion point (see below for details on analysis). Eye shape is defined as the sizeless variable described by regression between corneal diameter and axial length of the eye [2].

Eye size and shape are not, however, the only features of the eye that are expected to change with a shift from diurnality to nocturnality. For example, the outer nuclear layer, formed by the rod and cone nuclei, of nocturnal birds is generally much thicker and tends to have more rows than diurnal animals [5]. This is related to morphological differences in the photoreceptor types, with rods typically being longer than cones meaning the soma of the rod is usually located in the lower part of the ONL, and as in the mammalian retina, the nucleus of rods is smaller than cones [5]. Additionally, diurnal species need an increased number of horizontal, amacrine and ganglion cells to create the high resolving power needed for color vision in bright light conditions. Therefore, the inner nuclear and ganglion cell layers of diurnal species are thicker compared to nocturnal species [5]. We therefore compared the anatomical structure of the Kakapo retina with that of other parrots and other birds, all of which were processed in similar conditions. The eyes were dissected from the head and the anterior part and lens was removed prior to placing the tissue in 4% paraformaldehyde (PFA) for 30 minutes. The posterior eyecup was then washed and stored in 0.1 M phosphate buffer (PB) for 1 week. The eyes were cryoprotected in a series of 10% and 20% sucrose in PB solutions for 10 minutes each and then left overnight in a 30% sucrose PB solution. The posterior eyecup was embedded in TissueTek medium, frozen and cut perpendicular to the equator on a LEICA cryostat (Germany) at a thickness of 16–20 µm. Sections were mounted onto glycerine coated slides, stained with cresyl violet, dehydrated and coverslipped with DePeX. Images of central and peripheral areas of retina from Kakapo, Cockatoo, Barn Owl (*Tyto alba*), Chicken (*Gallus gallus domesticus*) and Eastern Rosella were obtained using a LEICA DC 500 camera and a 40× objective and 10× ocular lens. Central retina was defined as a 2 mm linear area around the optic nerve. Peripheral retina was 10 mm or more away from the optic nerve. Quantification consisted of measuring the total retinal thickness, the length of the outer and inner segment of the photoreceptor layer, and the outer, inner nuclear layer and ganglion cell layer thickness. Number of cells in the ganglion cell layer was counted in at least three different slides 100 µm apart in central and peripheral retina. The number of cells was averaged and expressed as GCL cells per mm linear retina.

### Data Analysis

To account for allometric effects on brain region volume and the morphometrics of the optic foramen and eye, all measurements were examined relative to multiple scaling variables. The data was log_10_ transformed prior to all analyses and the volume of each brain region was compared with brain volume minus the volume of the region of interest. For example, TeO volume was compared to the brain volume minus that of the TeO. In addition, we examined the relative size of the two telencephalic regions, E and Wulst, relative to telencephalic volume. For the eye measurements, we used brain volume and skull length, from the nasofrontal hinge to the caudal-most point of the brain case, as scaling variables. Finally, brain volume, skull length and foramen magnum area were used as the scaling variables for examining optic foramen diameter.

To determine if the Kakapo brain differed from other parrots, we performed least squares linear regressions using each of the dependent variables against the scaling variables outlined above. We then calculated 95% confidence intervals for these regression lines and screened for significant outliers by examining jackknife distances as calculated in JMP v. 5.1.2 (SAS Institute). To account for phylogenetic effects, we used the phylogeny of [24] with resolution of several genera provided by additional sources [79]–[81] and calculated phylogeny-corrected 95% confidence and prediction intervals using PDAP: PDTREE module [82] of Mesquite [83]. Because we reconstructed the phylogeny of all species from multiple sources, we used an arbitrary branch length model, which adequately standardized the independent contrasts [84], and could therefore be used to construct the ‘phylogeny-corrected’ confidence intervals.

## Supporting Information

Table S1 **The data used in all of the analyses.** ‘Orbits’ refers to the measurements taken for the degree of orbital convergence (‘Deg’), which is in degrees, and the sample size (‘n’) is provided for each species. ‘Eye Measurements’ were all taken from Ritland (1982) and are as follows: ‘CD’- corneal diameter (mm), ‘AL’ – axial length (mm), ‘TD’ – transverse orbital diameter (mm). ‘Skull Measurements’ were made from specimens at the National Museum of Natural History (Washington, DC) (samples are indicated under the ‘n’) and are as follows: ‘FM’ – foramen magnum area (mm^2^), ‘OF’ – maximum optic foramen diameter (mm), ‘EV’ – endocranial volume (mm^3^), and ‘HL’ – head length (mm). ‘Brain Volumetrics’ are the brain measurements made from serially sectioned brains, supplemented by four species taken from the literature (sample sizes are provided under the ‘n’). The brain regions are as follows: ‘Brain’ – total brain volume (mm^3^), ‘T’ – telencephalon (mm^3^), ‘W’ – Wulst (mm^3^), ‘E’ – entopallium (mm^3^), ‘nRt’ – nucleus rotundus (mm^3^) and ‘TeO’ – optic tectum (mm^3^). The values for the Kakapo (*Strigops habroptilus*) are highlighted in bold. ^1^Brain data from: Fernandez P, Carezzano F, Bee De Speroni N (1997) Analisis cuantitativo encefalico e indices cerebrales en Aratinga acuticaudata y Myiopsitta monachus de Argentina (Aves: Psittacidae). Rev Chil Hist Nat 70: 269–275. ^2^Brain data from: Boire D (1989) Comparaison quantitative de l'encephale de ses grades subdivisions et de relais visuals, trijumaux et acoustiques chez 28 especes. PhD Thesis, Universite de Montreal, Montreal.
(DOC)
