# Supplementary material for: Anatomical Specializations for Nocturnality in a Critically Endangered Parrot, the Kakapo (Strigops habroptilus)
Source: PLoS One. 2011 Aug 10;6(8):e22945. doi: 10.1371/journal.pone.0022945 (PMC3157909; doi:10.1371/journal.pone.0022945)
Supplement: Table S1 — The data used in all of the analyses. ‘Orbits’ refers to the measurements taken for the degree of orbital convergence (‘Deg’), which is in degrees, and the sample size (‘n’) is provided for each species. ‘Eye Measurements’ were all taken from Ritland (1982) and are as follows: ‘CD’- corneal diameter (mm), ‘AL’ – axial length (mm), ‘TD’ – transverse orbital diameter (mm). ‘Skull Measurements’ were made from specimens at the National Museum of Natural History (Washington, DC) (samples are indicated under the ‘n’) and are as follows: ‘FM’ – foramen magnum area (mm2), ‘OF’ – maximum optic foramen diameter (mm), ‘EV’ – endocranial volume (mm3), and ‘HL’ – head length (mm). ‘Brain Volumetrics’ are the brain measurements made from serially sectioned brains, supplemented by four species taken from the literature (sample sizes are provided under the ‘n’). The brain regions are as follows: ‘Brain’ – total brain volume (mm3), ‘T’ – telencephalon (mm3), ‘W’ – Wulst (mm3), ‘E’ – entopallium (mm3), ‘nRt’ – nucleus rotundus (mm3) and ‘TeO’ – optic tectum (mm3). The values for the Kakapo (Strigops habroptilus) are highlighted in bold. 1Brain data from: Fernandez P, Carezzano F, Bee De Speroni N (1997) Analisis cuantitativo encefalico e indices cerebrales en Aratinga acuticaudata y Myiopsitta monachus de Argentina (Aves: Psittacidae). Rev Chil Hist Nat 70: 269–275. 2Brain data from: Boire D (1989) Comparaison quantitative de l'encephale de ses grades subdivisions et de relais visuals, trijumaux et acoustiques chez 28 especes. PhD Thesis, Universite de Montreal, Montreal. (DOC) [file pone.0022945.s001.doc]

|  | **Orbits** | | **Eye Measurements** | | | **Skull Measurements** | | | | | **Brain Volumetrics** | | | | | | |
| --- | --- | --- | --- | --- | --- | --- | --- | --- | --- | --- | --- | --- | --- | --- | --- | --- | --- |
| **Species** | **n** | **Deg** | **CD** | **AL** | **TD** | **n** | **FM** | **OF** | **EV** | **HL** | **n** | **Brain** | **T** | **W** | **E** | **nRt** | **TeO** |
| *Agapornis cana* | 3 | 15.73 | 3.4 | 5.5 | 7.2 | 3 | 9.34 | 1.68 | 1167 | 19.05 |  |  |  |  |  |  |  |
| *Agapornis fischeri* | 1 | 14.67 | 4.45 | 7.6 | 8.9 | 3 | 12.16 | 1.93 | 2000 | 23.18 |  |  |  |  |  |  |  |
| *Agapornis personata* | 3 | 15.15 |  |  |  | 3 | 12.19 | 1.88 | 1870 | 22.67 | 1 | 2824.32 | 2069.65 | 204.54 |  |  | 82.57 |
| *Agapornis roseicollis* | 3 | 10.34 |  |  |  | 3 | 12.17 | 1.69 | 1860 | 22.84 | 1 | 2020.27 | 1454.88 | 194.32 | 3.1 | 2.785 | 79.74 |
| *Alisterus scapularis* | 3 | 14.12 | 7 | 12.4 | 14.4 | 2 | 21.02 | 2.86 | 4480 | 33.9 | 3 | 4794.13 | 3271.46 | 510.08 | 16.42 | 6.318 | 202.14 |
| *Amazona aestiva* | 2 | 15.94 |  |  |  |  |  |  |  |  | 1 | 7954.63 | 5672.01 | 760.1 | 21.31 | 11.213 | 273.47 |
| *Amazona albifrons* | 4 | 9.73 | 6.8 | 12.2 | 14.3 | 2 | 27.1 | 2.71 | 5200 | 35.33 |  |  |  |  |  |  |  |
| *Amazona imperialis* |  |  | 8.4 | 14.1 | 17.2 | 3 | 30.98 | 3.6 | 8290 | 43.3 |  |  |  |  |  |  |  |
| *Amazona ochrocephala* | 3 | 14.41 | 9.4 | 15.3 | 17.7 |  |  |  |  |  |  |  |  |  |  |  |  |
| *Anodorhynchus hyacinthus* | 4 | 7.04 | 10.1 | 16.8 | 19.8 | 3 | 35.47 | 3.52 | 8650 | 44.01 |  |  |  |  |  |  |  |
| *Aprosmictus erythropterus* | 2 | 14.22 | 6.3 | 10.5 | 12.7 | 2 | 63.47 | 4.35 | 25625 | 76.77 |  |  |  |  |  |  |  |
| *Ara ararauna* | 3 | 9.57 | 8.8 | 15.8 | 19 | 3 | 19.11 | 2.61 | 3733 | 31.28 |  |  |  |  |  |  |  |
| *Ara militaris* | 3 | 11.39 | 8.8 | 16 | 19.3 | 3 | 57.57 | 3.89 | 19000 | 62.57 |  |  |  |  |  |  |  |
| *Ara severa* | 2 | 6.36 | 7.5 | 12.5 | 16.5 | 3 | 62.28 | 4.21 | 19083 | 59.43 |  |  |  |  |  |  |  |
| *Aratinga acuticaudata*^1^ | 2 | 12.71 |  |  |  | 2 | 31.45 | 3.39 | 9525 | 44.22 | 1 | 5222 | 4325.91 | 240.73 |  |  |  |
| *Aratinga leucophthalmus* | 2 | 12.15 | 6.4 | 10.9 | 13.25 | 1 | 17.7 | 2.62 | 5250 | 35.06 |  |  |  |  |  |  |  |
| *Aratinga pertinax* | 4 | 9.33 | 5.3 | 9.4 | 11 | 3 | 22.88 | 3.01 | 5700 | 34.66 |  |  |  |  |  |  |  |
| *Barnardius zonarius* | 1 | 14.08 | 6.45 | 10.5 | 12.75 | 3 | 16.84 | 2.28 | 3433 | 28.73 |  |  |  |  |  |  |  |
| *Bolbopsittacus lunulatus* |  |  | 5.9 | 8.1 | 11.2 |  |  |  |  |  |  |  |  |  |  |  |  |
| *Bolborhynchus lineola* |  |  | 5 | 7.7 | 9.3 | 2 | 16.59 | 2.35 | 2650 | 27.19 |  |  |  |  |  |  |  |
| *Brotogeris versicolurus* | 2 | 14.24 | 4.25 | 8.3 | 10.05 | 2 | 12.94 | 1.84 | 1950 | 24.47 |  |  |  |  |  |  |  |
| *Cacatua alba* | 1 | 18.03 | 9 | 15.3 | 19.5 | 3 | 13.81 | 2.07 | 2283 | 25.09 |  |  |  |  |  |  |  |
| *Cacatua ducorpsii* | 1 | 17.69 | 6.3 | 12.4 | 14.5 | 3 | 54.07 | 4.2 | 14167 | 49.99 |  |  |  |  |  |  |  |
| *Cacatua galerita* | 3 | 17.96 | 7.5 | 14 | 17.2 | 1 | 37.95 | 3 | 8300 | 41.78 | 1 | 14515 | 11292.48 |  | 47.59 | 9.58 | 322.11 |
| *Cacatua haematuropygia* | 2 | 15.92 | 7.3 | 12.6 | 15.2 | 2 | 47.04 | 3.87 | 13875 | 49.07 |  |  |  |  |  |  |  |
| *Cacatua leadbeateri* | 1 | 10.12 | 7.1 | 12.2 | 15.2 | 1 | 37.87 | 3.7 | 8800 | 39.53 |  |  |  |  |  |  |  |
| *Cacatua sanguinea* |  |  | 5.2 | 12.2 | 14.1 | 3 | 30.34 | 3.05 | 8817 | 42.52 |  |  |  |  |  |  |  |
| *Callocephalon fimbriatum* | 3 | 16.22 | 7.1 | 12.3 | 15 | 3 | 25.78 | 3.38 | 6430 | 36.45 |  |  |  |  |  |  |  |
| *Calyptorhynchus banskii* | 2 | 9.55 |  |  |  | 2 | 32.24 | 2.84 | 9025 | 39.25 |  |  |  |  |  |  |  |
| *Calyptorhynchus funereus* | 2 | 7.84 | 9.3 | 16.6 | 19 | 3 | 30.17 | 2.91 | 7450 | 38.84 | 1 | 16111 | 12823.58 | 2036.46 | 42.23 | 11.947 | 309.66 |
| *Calyptorhynchus magnificus* |  |  | 9.1 | 15.8 | 18.2 |  |  |  |  |  |  |  |  |  |  |  |  |
| *Chalcopsitta atra* | 1 | 19.59 | 6.1 | 10.5 | 13.2 | 1 | 51.72 | 3.08 | 13000 | 49.78 |  |  |  |  |  |  |  |
| *Charmosyna papou* | 3 | 8.47 | 5.2 | 9.3 | 11.1 | 2 | 31.8 | 2.97 | 5325 | 38.59 |  |  |  |  |  |  |  |
| *Charmosyna placentis* | 1 | 11.42 | 4.2 | 6.5 | 8.4 | 3 | 19.55 | 2.44 | 3417 | 31.37 |  |  |  |  |  |  |  |
| *Charmosyna rubrigularis* |  |  | 4.6 | 7 | 8.5 |  |  |  |  |  |  |  |  |  |  |  |  |
| *Coracopsis vasa* | 2 | 8.59 | 8.5 | 14.6 | 17.7 |  |  |  |  |  |  |  |  |  |  |  |  |
| *Cyanoliseus patagonus* | 3 | 15.26 | 7 | 11.4 | 13.4 | 2 | 36.04 | 3.36 | 8600 | 45.54 |  |  |  |  |  |  |  |
| *Cyanoramphus novaezelandiae* | 2 | 20.97 |  |  |  | 3 | 26.78 | 2.85 | 8117 | 38.52 |  |  |  |  |  |  |  |
| *Cyanoramphus unicolor* |  |  | 6.1 | 9.3 | 11.5 | 1 | 20.12 | 2.55 | 4150 | 32.28 |  |  |  |  |  |  |  |
| *Deroptyus accipitrinus* | 3 | 11.97 | 7.8 | 13.2 | 16.8 |  |  |  |  |  |  |  |  |  |  |  |  |
| *Eclectus roratus* | 2 | 9.73 | 8.3 | 13.8 | 16.8 | 3 | 26.92 | 3.2 | 7767 | 38.95 | 2 | 6248.07 | 4583.16 | 701.68 | 23.31 | 7.96 | 221.1 |
| *Eolophus roseicapillus* | 5 | 33.52 | 5.6 | 12.1 | 13 | 2 | 29.17 | 3.19 | 7360 | 42.17 | 2 | 6666.15 | 4908.67 | 675.89 | 22.26 | 7.103 | 204.59 |
| *Eos bornea* | 3 | 10.35 | 6.3 | 10.4 | 12.2 | 3 | 23.17 | 2.74 | 5317 | 33.27 |  |  |  |  |  |  |  |
| *Eos cyanogenia* |  |  | 5.8 | 10 | 12.5 | 3 | 25.04 | 2.77 | 4700 | 35.77 |  |  |  |  |  |  |  |
| *Eos squamata* |  |  |  |  |  | 2 | 22.19 | 2.72 | 4850 | 35.43 |  |  |  |  |  |  |  |
| *Eunymphicus cornutus* |  |  | 6.7 | 11.5 | 13.2 | 3 | 19.99 | 2.45 | 3950 | 32.56 |  |  |  |  |  |  |  |
| *Forpus passerinus* | 3 | 14.91 | 3.8 | 6.3 | 7.5 | 2 | 19.48 | 2.73 | 3325 | 29.5 |  |  |  |  |  |  |  |
| *Geoffroyus geoffroyi* |  |  | 7.6 | 11.9 | 14.6 | 3 | 8.95 | 1.48 | 1150 | 19.55 |  |  |  |  |  |  |  |
| *Geoffroyus heteroclitus* | 2 | 7.55 |  |  |  | 3 | 24.29 | 3.05 | 4000 | 33.56 |  |  |  |  |  |  |  |
| *Glossopsitta concinna* | 3 | 10.75 | 5.2 | 8.1 | 10.1 | 2 | 15.07 | 2.26 | 2870 | 28.5 | 3 | 3159.2 | 2272.74 | 358.43 | 8.6 | 5.294 | 98.76 |
| *Glossopsitta porphyrocephala* |  |  | 4.3 | 6.8 | 8.3 |  |  |  |  |  |  |  |  |  |  |  |  |
| *Glossopsitta pusilla* |  |  | 3.8 | 6.6 | 8 |  |  |  |  |  |  |  |  |  |  |  |  |
| *Graydidasculus brachyurus* | 1 | 10.57 | 6.1 | 11.6 | 13 | 1 | 31.18 | 2.78 | 5300 | 35.78 |  |  |  |  |  |  |  |
| *Lathamus discolor* |  |  | 4.9 | 8.05 | 9.9 | 1 | 19.67 | 2.49 | 2250 | 24.97 |  |  |  |  |  |  |  |
| *Loriculus berrylinus* |  |  | 4.3 | 6.8 | 9.3 |  |  |  |  |  |  |  |  |  |  |  |  |
| *Lorius garrulous* | 3 | 12.31 | 6.1 | 10.2 | 12.8 | 3 | 28.57 | 3.08 | 5067 | 38.17 |  |  |  |  |  |  |  |
| *Lorius lorry* |  |  | 6.1 | 10.3 | 12.8 | 3 | 29.72 | 3.11 | 5167 | 38.81 |  |  |  |  |  |  |  |
| *Melopsittacus undulatus*^2^ | 1 | 33.79 | 3.4 | 6.9 | 7.5 | 3 | 10.24 | 1.58 | 1500 | 19.77 | 1 | 1220 | 825.12 | 84.35 | 3.93 | 1.882 | 59.64 |
| *Micropsitta bruijnii* |  |  | 2.9 | 5.3 | 6.8 |  |  |  |  |  |  |  |  |  |  |  |  |
| *Micropsitta finschii* | 1 | 12.67 | 3.15 | 5.6 | 6.95 | 1 | 6.95 | 1.45 |  |  |  |  |  |  |  |  |  |
| *Micropsitta pusio* |  |  | 3.55 | 5.7 | 7.45 |  |  |  |  |  |  |  |  |  |  |  |  |
| *Myiopsitta monachus*^1^ | 1 | 30.33 | 5.4 | 8.7 | 10.2 | 3 | 15.92 | 2.45 | 4080 | 30.31 | 1 | 3697 | 2733.19 | 253.61 |  |  |  |
| *Nandayus nenday* |  |  | 5.1 | 9.9 | 11.8 | 1 | 17.94 | 2.4 | 4750 | 31.14 |  |  |  |  |  |  |  |
| *Neophema splendida* |  |  | 4.35 | 6.8 | 9.4 | 3 | 9.41 | 1.93 | 1333 | 21.73 |  |  |  |  |  |  |  |
| *Neopsephotus bourkii* |  |  | 4.7 | 7.5 | 9.65 | 2 | 9.21 | 1.81 | 1250 | 22.06 | 1 | 1213 | 834.24 | 118.23 | 3.08 |  | 56.42 |
| *Neopsittacus musschenbroekii* |  |  | 4.8 | 7.6 | 9.5 |  |  |  |  |  |  |  |  |  |  |  |  |
| *Nestor meridionialis* | 1 | 8.86 | 7.9 | 12.2 | 14.95 | 1 | 31.34 | 3.3 | 13000 | 52.08 |  |  |  |  |  |  |  |
| *Nestor notabilis* | 3 | 20.48 | 8.8 | 14.3 | 17 | 5 | 42.64 | 3.79 | 17150 | 51.89 | 1 | 13388.03 | 12420.54 | 1505.48 | 23.73 | 9.033 | 319.99 |
| *Nymphicus hollandicus* | 3 | 19.55 | 4.97 | 8.8 | 10.73 | 3 | 13.76 | 2.52 | 2390 | 25.09 | 2 | 2339.29 | 1676.78 | 250.61 | 11.37 | 4.195 | 80.82 |
| *Opopsitta diophthalmica* |  |  | 4.9 | 8.2 | 9.5 |  |  |  |  |  |  |  |  |  |  |  |  |
| *Oreopsittacus arfaki* |  |  | 3.3 | 5.8 | 6.95 | 2 | 9.25 | 1.61 | 1150 | 21.18 |  |  |  |  |  |  |  |
| *Pezoporus wallicus* | 1 | 11.65 | 5.4 | 11 | 12.6 |  |  |  |  |  |  |  |  |  |  |  |  |
| *Phigys solitarius* |  |  | 4.9 | 8.2 | 10.3 |  |  |  |  |  |  |  |  |  |  |  |  |
| *Pionites melanocephala* |  |  | 7.1 | 11.8 | 14.4 | 3 | 22.85 | 2.66 | 5083 | 36.8 |  |  |  |  |  |  |  |
| *Pionopsitta pileata* |  |  | 5.3 | 9.8 | 11.6 |  |  |  |  |  |  |  |  |  |  |  |  |
| *Pionus chalcopterus* |  |  | 7.7 | 12.8 | 15.8 | 2 | 25.71 | 3.25 | 6125 | 36.2 |  |  |  |  |  |  |  |
| *Pionus menstruus*^2^ | 1 | 24.97 | 7.5 | 12.5 | 16.4 | 3 | 29.58 | 3.28 | 5700 | 37.52 | 1 | 5283 | 3851.82 | 408.63 | 16.12 | 9.297 | 257.95 |
| *Platycercus adscitus* |  |  | 5.2 | 9.6 | 11 | 3 | 17.05 | 2.53 | 3217 | 28.85 |  |  |  |  |  |  |  |
| *Platycercus elegans* |  | 32.82 |  |  |  | 2 | 17.88 | 2.64 | 3780 | 31.71 | 3 | 3822.37 | 2687.57 | 348.05 | 12.47 | 5.651 | 160.24 |
| *Platycercus eximius* |  |  |  |  |  | 3 | 14.08 | 3.55 | 3010 | 28.57 | 4 | 3258.48 | 2326.68 | 317.72 | 10.565 | 4.721 | 129.84 |
| *Poicephalus meyeri* |  |  | 6.75 | 10.7 | 12.9 | 3 | 22.32 | 2.63 | 4500 | 31.52 |  |  |  |  |  |  |  |
| *Poicephalus robustus* | 2 | 10.14 |  |  |  | 3 | 26.2 | 2.85 | 4900 | 33.77 |  |  |  |  |  |  |  |
| *Poicephalus senegalus* | 3 | 11.12 | 6.9 | 10.7 | 13.6 | 1 | 15.99 | 2.59 | 3200 | 26.69 |  |  |  |  |  |  |  |
| *Polytelis alexandrae* | 1 | 18.64 | 5.3 | 8.65 | 11.25 |  |  |  |  |  |  |  |  |  |  |  |  |
| *Polytelis swainsonii* | 2 | 38.29 |  |  |  |  |  |  |  |  | 2 | 3157.23 | 2163.2 | 287.02 |  | 4.13 | 134.88 |
| *Prioniturus lucionensis* |  |  | 6.05 | 10.95 | 12.85 | 2 | 56.53 | 3.74 | 20375 | 72.6 |  |  |  |  |  |  |  |
| *Probosciger aterrimus* |  |  | 10.6 | 17.6 | 21.3 | 2 | 24.66 | 3.37 | 5300 | 40.05 |  |  |  |  |  |  |  |
| *Prosopeia tabuensis* |  |  | 7.8 | 13.1 | 15.2 | 2 | 11.04 | 1.97 | 1970 | 24.76 |  |  |  |  |  |  |  |
| *Psephotus haematonotus* | 2 | 29.19 | 4.4 | 8.4 | 10.4 | 1 | 23.48 | 2.63 | 4200 | 34.68 | 2 | 1940.16 | 1402.55 | 174.2 | 10.39 | 3.437 | 73.47 |
| *Pseudeos fuscata* | 1 | 14.98 | 5.7 | 8.8 | 12.4 |  |  |  |  |  |  |  |  |  |  |  |  |
| *Psittacella brehmii* |  |  | 6 | 11 | 13 | 2 | 28.17 | 3.12 | 5540 | 40.53 |  |  |  |  |  |  |  |
| *Psittacula eupatria* |  |  |  |  |  |  |  |  |  |  | 1 | 6327.22 | 4942.37 |  | 19.304 |  | 160.94 |
| *Psittacula himalayana* |  |  | 5.5 | 9.6 | 12.5 | 3 | 19.01 | 2.41 | 3900 | 31.89 |  |  |  |  |  |  |  |
| *Psittacula krameri* | 1 | 24.03 | 5.6 | 9.8 | 11.8 |  |  |  |  |  | 1 | 4243.24 | 3269.62 | 565.67 |  |  |  |
| *Psittacula longicauda* |  |  | 6.5 | 10.3 | 12.6 | 3 | 38.45 | 2.98 | 9180 | 44.07 |  |  |  |  |  |  |  |
| *Psittacus erithacus* | 1 | 12.14 | 8.1 | 13.2 | 16 | 2 | 17.72 | 2.59 | 3025 | 27.7 | 1 | 6405.41 | 4726.89 | 668.55 | 15.49 | 7.412 | 155.14 |
| *Psittinus cyanurus* |  |  | 6 | 9.9 | 12.4 | 1 | 39.27 | 3.61 | 7800 | 50.82 |  |  |  |  |  |  |  |
| *Psittrichas fulgidus* |  |  | 8.3 | 13.9 | 17.6 | 2 | 17.72 | 2.7 | 3725 | 28.86 |  |  |  |  |  |  |  |
| *Purpuriecephalus spurious* | 1 | 18.19 | 6 | 10.6 | 12.9 | 1 | 12.25 | 1.8 | 2100 | 24.24 |  |  |  |  |  |  |  |
| *Pyrrhura leucotis* | 3 | 14.7 | 4.6 | 7.4 | 9.4 |  |  |  |  |  |  |  |  |  |  |  |  |
| *Pyrrhura molinae* | 2 | 5.59 | 4.75 | 8.25 | 10.35 |  |  |  |  |  | 1 | 4673.75 | 3123.51 | 497.19 |  | 8.259 | 232.93 |
| *Rhynchopsitta pachyrhyncha* |  |  | 7.2 | 11.3 | 14 | 2 | 49.26 | 1.99 | 15250 | 52.705 |  |  |  |  |  |  |  |
| ***Strigops habroptilus*** | **2** | **59** | **9** | **13.45** | **15.55** | **2** | **29.12** | **3.18** | **6250** | **39.24** | **1** | **14768** | **12420.54** | **1754.89** | **12.45** | **4.303** | **122.75** |
| *Tanygnathus lucionensis* |  |  | 7.9 | 13 | 15.2 | 3 | 19.53 | 2.32 | 3117 | 29.14 |  |  |  |  |  |  |  |
| *Tanygnathus sumatranus* | 2 | 14.04 |  |  |  | 3 | 21.04 | 2.77 | 3660 | 33.14 |  |  |  |  |  |  |  |
| *Trichoglossus chlorolepidotus* |  |  | 5.2 | 8.7 | 10.7 | 2 | 22 | 3.08 | 4275 |  |  |  |  |  |  |  |  |
| *Trichoglossus haematodus* | 1 | 36.75 | 5.75 | 9.95 | 12.05 | 1 | 13.57 | 2.09 |  | 25.72 | 2 | 3727.83 | 2726.62 | 411.25 | 10.089 | 4.889 | 123.42 |
| *Triclaria malachitacea* |  |  | 7.1 | 11.6 | 13.6 |  |  |  |  |  |  |  |  |  |  |  |  |
| *Vini australis* | 3 | 16.78 | 4.4 | 7.2 | 8.6 |  |  |  |  |  |  |  |  |  |  |  |  |
| *Vini kuhlii* |  |  | 3.9 | 6.3 | 8.1 |  |  |  |  |  |  |  |  |  |  |  |  |
| *Vini peruviana* |  |  | 3.5 | 5.6 | 7.2 |  |  |  |  |  |  |  |  |  |  |  |  |
